# Polypyrrole Coatings as Possible Solutions for Sensing and Stimulation in Bioelectronic Medicines

**DOI:** 10.3390/bios15060366

**Published:** 2025-06-06

**Authors:** Cristian Sevcencu, Izabella Crăciunescu, Alin-Alexandru Andrei, Maria Suciu, Sergiu Macavei, Lucian Barbu-Tudoran

**Affiliations:** 1 National Institute for Research and Development of Isotopic and Molecular Technologies (INCDTIM), 400293 Cluj-Napoca, Romania; cristian.sevcencu@itim-cj.ro (C.S.); aandrei@itim-cj.ro (A.-A.A.); maria.suciu@itim-cj.ro (M.S.); macavei.sergiu@itim-cj.ro (S.M.); lucian.barbu@itim-cj.ro (L.B.-T.); 2 Doctoral School in Integrative Biology, Faculty of Biology and Geology, Babes-Bolyai University, 400006 Cluj-Napoca, Romania; 3 Electron Microscopy Center “C. Craciun”, Faculty of Biology and Geology, Babes-Bolyai University, 400006 Cluj-Napoca, Romania

**Keywords:** bioelectronic medicine, polypyrrole, action potentials, recording, stimulation

## Abstract

Bioelectronic medicines record biological signals and provide electrical stimulation for the treatment of diseases. Advanced bioelectronic therapies require the development of electrodes that match the softness of the implanted tissues, as the present metal electrodes do not meet this condition. The objective of the present work was to investigate whether the electroconductive polymer polypyrrole (PPy) could be used for fabricating such electrodes, as PPy is several orders softer than metals. For this purpose, we here investigated if electrodes made using coatings and films of PPy doped with naphthalin-2-sulfonic acid (PPy/N) are capable to record and elicit by stimulation cardiac monophasic action potentials (MAPs) and if PPy/N is also biocompatible. The results of this study showed that the tested PPy/N electrodes are capable of recording MAPs almost identical to the MAPs recorded with platinum electrodes and eliciting stimulation-evoked MAPs almost identical to the spontaneous MAPs. In addition, we show here that the cell cultures that we used for biocompatibility tests grew in a similar manner on PPy/N and platinum substrates. We, therefore, conclude that PPy/N coatings and films have recording and electrical stimulation capabilities that are similar to those of platinum electrodes and that PPy/N substrates are as biocompatible as the platinum substrates.

## 1. Introduction

Besides classical bioelectronic therapies that were approved decades ago and presently operate in numerous patients (e.g., for cardiac pacing [1] and treatment of Parkinson’s disease [2], refractory epilepsy [3] or chronic pain [4]), new bioelectronic therapies, such as for treatment of hypertension [5], paralysis [6], inflammatory diseases [7], and even psychiatric disorders [8] are in development and could be available in the future. While most of the present bioelectronic therapies lack closed-loop control abilities and therefore are not adaptable in real time to the ever-changing condition of the patient, the development of closed-loop bioelectronic therapies adaptable in real time to the actual patient’s needs is in progress [4,9,10]. Moreover, some of such closed-loop bioelectronic therapies are even meant to use just one electrode for both the delivery of stimulation and for recording biological signals that are used as closed-loop variables for the procedure [11]. Although a number of achievements have been reported in those directions [9,10], such next-generation closed-loop bioelectronic therapies are yet “in their infancy” [2] as biomedical and technological challenges must still be overcome in order to make them safe and fully operative [11].

One of such challenges is the development of electrodes with stable recording and stimulation capabilities, as the recording and stimulation capabilities of the present electrodes are unstable over the months and years of implantation [12,13,14]. One reason for that instability is the mechanical mismatch between the stiffness of the current metal/silicone electrodes and the softness of the living tissue [11,12]. This mismatch leads to electrode movements relative to the tissue during tissue motions, damages the host tissue and/or the electrode structure, and triggers encapsulation of the electrode in scar tissue, which impedes the transmission of stimulation pulses and biological signals between the electrode and implanted tissue [11,12]. As the envisioned closed-loop technologies cannot properly operate in such conditions, the development of such technologies requires the design of electrodes that better match the softness of the living tissue [11,12].

One possible solution for fabricating such electrodes could be by using conductive polymers for this purpose. Indeed, while having low impedance and the capability to conduct both electronic and ionic charges, such polymers are also several orders softer than metals and biocompatible, which makes them suitable for such an application [15]. Although implantable electrodes made from conductive polymers are not available yet for clinical bioelectronic therapies, several polymers, such as polyaniline, poly(3,4-ethylenedioxythiophene), poly(3,4-ethylenedioxythiophene) polystyrene sulfonate or polypyrrole seem suitable from this perspective as studies have shown that their deposition on metallic electrodes increases the bioelectronic capabilities of the latter [12,15].

One polymer that could, in principle, be used for fabricating electrodes softer than those made from metals is polypyrrole (PPy), as studies have shown that PPy has excellent conductivity and biocompatibility [16,17,18]. However, PPy also has two limitations that must be overcome in order to make it suitable for fabricating electrodes for chronic use. Those limitations are the brittleness of PPy [18] and its declining conductivity in aqueous environments due to de-doping processes [19]. One way to cope with the brittleness of PPy is by depositing PPy coatings on substrates with better mechanical properties and using such composites for making electrodes. Meanwhile, de-doping of such PPy coatings could be avoided by doping the PPy with large molecule dopants, which are less mobile in aqueous solutions and thus more stable.

To date, several studies have shown that PPy coatings deposited on platinum, gold, and iridium oxide electrodes, which are the typical electrodes in present bioelectronic therapies [3], are able to mediate the recording of biological signals and the electrical stimulation of cultured cells and even to improve the biocompatibility and conductivity of such metallic electrodes [20,21,22,23,24]. However, because in such an electrode configuration, the substrate for the PPy coating is still a metallic component, the mismatch between the stiffness of such an electrode and the tissue softness would be the same as for the metal electrodes.

As a first step towards the fabrication of electrodes by depositing PPy coatings on non-metallic substrates softer than metals, we here investigated whether PPy coatings doped with naphthalin-2-sulfonic acid (i.e., a large molecule dopant) could be used to record biological signals and stimulate living tissues. In order to compare the recording capabilities of coatings of PPy doped with naphthalin-2-sulfonic acid (PPy/N) with those of platinum (Pt) electrodes, spontaneous cardiac action potentials were simultaneously recorded with electrodes made from nylon filaments coated with PPy/N and Pt electrodes. In order to investigate the capability of a PPy/N coating to stimulate living tissues, we also investigated here if PPy/N could be used to elicit, by electrical stimulation-evoked cardiac action potentials. Because the biocompatibility of PPy/N has not been studied yet, we also investigated the biocompatibility of this material using cell culture techniques.

## 2. Materials and Methods

### 2.1. Preparation and Conductivity of PPy/N Coatings and Films

Polymerization of the pyrrole monomer can be achieved by oxidative chemical deposition and electrochemical techniques. In this study, both approaches were used to synthesize polymeric materials with properties optimized for the targeted application.

**Figure 1:**
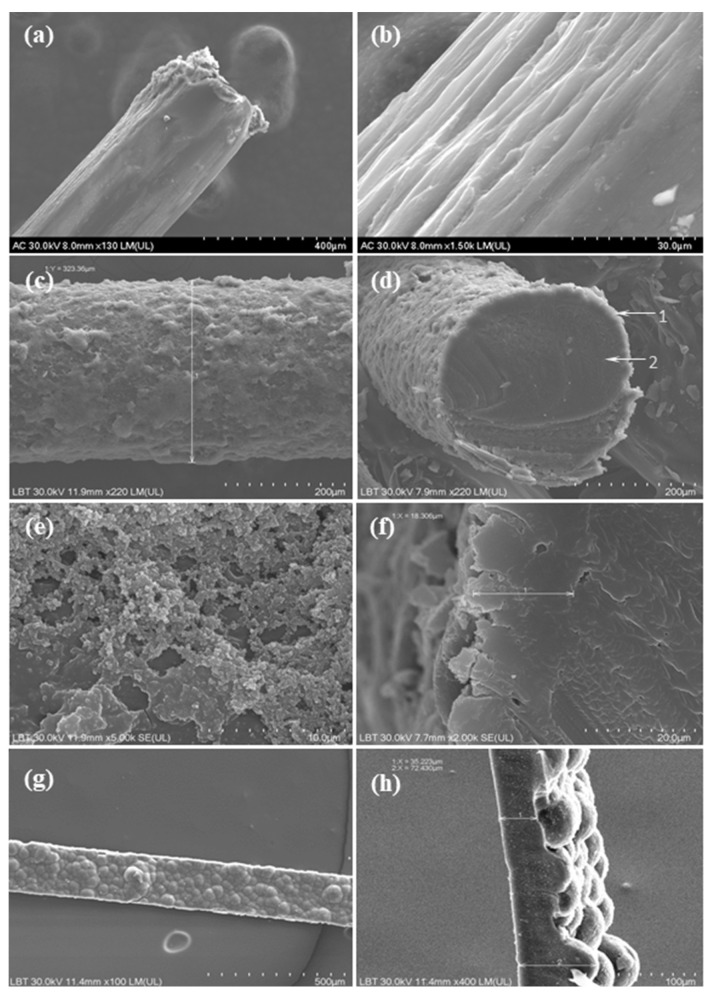
Electron microscopy images of uncoated (**a**,**b**) and PPy/N coated nylon filaments (**c**–**f**), and of the PPy/N strips (**g**,**h**) that were used to fabricate the recording and stimulation electrodes, respectively.

*PPy/N coatings on nylon filaments.* PPy/N coatings deposited on nylon filaments were prepared through chemical oxidative polymerization, i.e., a chain-growth process which was facilitated by the doping agent naphthalin-2-sulfonic acid (N) and the oxidizing agent ammonium persulfate (APS). Eight-centimeter-long nylon filaments with a diameter of 300 µm were initially cleaned in an ultrasonic bath using an alcohol-based solution. To improve the adhesion of the PPy coating, the surface of the filaments was roughened through an abrasive treatment in order to disrupt their smooth texture and thus enhance the adsorption of the dopant solution. Subsequently, the filaments were immersed for 60 min in a 0.15 M aqua solution of an N doping agent to allow the N molecules to adhere to their surface. The pyrrole monomer was then added to that solution at a concentration of 0.6 M while gently magnetic stirring to ensure a proper homogenization of the solution. To initiate the polymerization reaction, the APS was added dropwise to the solution to a concentration of 2 M. The reaction was carried out at room temperature for 2 h under continuous gentle stirring. During this process, a uniform layer of PPy doped with N (PPy/N) was deposited on the nylon filaments. The nylon filaments coated with PPy/N were then washed with alcohol, dried, and stored in a dark environment until used for determining the conductivity of the PPy/N coating and fabrication of recording PPy/N electrodes. Figure 1 presents electron microscopy images of nylon filaments before (Figure 1a,b) and after being coated with PPy/N (Figure 1g,h).

*Free-standing PPy/N films.* Free-standing PPy/N films were fabricated using electrochemical polymerization, which was carried out in an acetonitrile electrolyte solution containing pyrrole monomer at a concentration of 0.1 M and the N doping agent at a concentration of 0.05 M. A two-electrode setup was employed using stainless steel electrodes (3 × 3 cm) as both working and counter electrodes. The polymerization process was controlled potentiostatically at 0.8 V for two hours, during which a uniform layer of PPy/N was deposited on the electrode surface. The PPy/N film was detached from the working electrode, rinsed with alcohol, dried, and cut into 200 µm wide strips using a sharp razorblade. The strips were then stored in the dark until used for determining their conductivity and fabrication of stimulation PPy/N electrodes. Electron microscopy images of such PPy/N strips are presented in Figure 1g,h.

*Conductivity of the PPy/N coatings and strips.* The conductivity (*σ*) of the PPy/N coatings and strips was calculated based on their resistivity (*ρ*) as *σ =* 1*/ρ*. The resistivity of the samples was calculated as *ρ = Vs/Il*, where *V* was the voltage generated by a current *I* that passed through the sample with the cross-section surface *s* and *l* the distance between the probes used to measure *V*. While *s* was calculated based on the SEM images (Figure 1), *V* was measured using the collinear 4-probe technique described by Schroder [25]. In our setup, the 4 probes of the measuring system were placed in contact with the samples at an equal distance *l* of 10 mm in between. Using a 2450 SourceMeter (Keithley Instruments, Tektronix, Bracknell, UK), a 200 µA DC current *I* was supplied to the 2 outer probes, and *V* was measured between the 2 inner probes of the measuring system. The values of *l*, *I*, *s*, and *V* were then used to calculate *ρ* and *σ* as stated above.

### 2.2. Recording and Stimulation Experiments

*The experimental model.* The cardiac action potentials investigated in this study were recorded in the form of monophasic action potentials (MAPs) from the left ventricle of eighteen 13-day-old chick embryos by adapting to this animal model the method that Knollmann et al. used to record mouse MAPs [26]. While having cardiac action potentials identical to those of mammals [27,28,29], the chick embryos are cheap, readily available in large numbers, and free of ethical constraints until including day 13 of incubation, when they are large enough for cardiac manipulation, but still incapable to feel pain [30,31,32]. As such, the animal model used in the present work fully complies with the European Directive 2010/63/EU, in general, and, in particular, with regard to the rule of replacing mammals as laboratory animals with animals having the lowest or no capacity to experience pain. Moreover, in accordance with the same European Directive 2010/63/EU, no ethical approval is required for using 13-day-old chick embryos in animal experimental studies [30,31,32].

*Surgical procedures.* The eggs were placed in support and maintained at a constant temperature of 38 ± 1 °C throughout the experiment using a Lauda heating system (Hugo Sachs Elektronik, March-Hugstetten, Germany). In order to access the embryo, the eggs were placed in a horizontal position, and, protecting the integrity of the air chamber, the upper part of the shell was removed. The vitelline membrane was then also removed, and the embryo was positioned ventral side up. To reduce the embryo’s movements, its neural tube was sectioned at the base of the cranium. To expose the heart, the ventral part of the ribcage and pericardium were removed.

**Figure 2:**
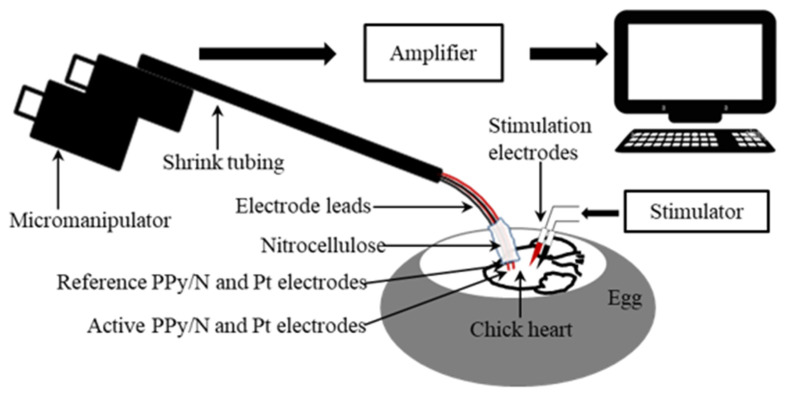
The experimental setup used to simultaneously record monophasic action potentials (MAPs) from the surface of chick embryo ventricles and to induce by electrical stimulation evoked MAPs.

*Recording of MAPs.* In order to compare the recording capabilities of PPy/N coatings vs. Pt electrodes, spontaneous MAPs were simultaneously recorded from the ventricle of nine chick embryo hearts (n = 9) with two pairs of electrodes consisting of an active and an indifferent electrode each. The electrodes from the first pair consisted of two 5 mm long segments of PPy/N coated nylon filaments (Figure 1a–d), and those from the second pair of two segments of Pt wires with the same length. The electrodes had similar diameters, i.e., around 320 µm for the PPy/N coated filaments (Figure 1a–d) and 300 µm for the Pt wires. The proximal ends of the electrodes were connected to 15 cm long Teflon-coated stainless steel leads (150/300 µm in diameter) using silver epoxy (Agar Scientific Ltd., Rotherham, UK). Except for their distal tips, which were completely coated with PPy/N in the case of the PPy/N electrodes, the electrodes were insulated with an approximately 150 µm thick layer of nitrocellulose that also covered the electrode-lead silver epoxy bond. The four electrodes were then grouped together and included in a common layer of nitrocellulose in such a manner that the distal tips of the active PPy/N and Pt electrodes were located 1 mm in front of their reference counterparts as indicated by Knollmann et al. [26], and in the same horizontal plane. The four leads were then twisted together, included in shrink tubing, curved downwards at approximately 60° at the electrode end, and mounted in a micromanipulator. The opposite free ends of the leads were connected to the headstages of the amplification system (Figure 2).

During the recordings, the tips of the active PPy/N and Pt electrodes were gently pressed on the left ventricle of the hearts and maintained in stable contact with the ventricle throughout the cardiac cycle through the spring force exerted by the curved region of the electrode leads (Figure 2). Attention was focused on also maintaining the indifferent PPy/N and Pt electrodes submerged in the albumen. The MAP signals were amplified using 2 low-noise headstages and an Iso-DAM 8A amplifier (World Precision Instruments, Sarasota, FL, USA) (10× and 10,000×, respectively) low pass (0.1 Hz) filtered, and fed to a computer through a Power 3 1401 DAQ operated by Spike 2, version 7.10 software (Cambridge Electronic Design Ltd., Cambridge, UK).

*Stimulation experiments.* The stimulation experiments were also performed by adapting the method of Knollmann et al. [26], who used Pt wire electrodes inserted in the mouse ventricle to stimulate the ventricular myocardium and so induce electrically evoked MAPs. In order to investigate whether such evoked MAPs could also be induced by stimulation delivered through PPy/N electrodes, we inserted one pair of PPy/N electrodes (i.e., cathode and anode) in the left ventricle of chick embryos and tested the effect of stimulation through those electrodes. Because the ventricles in 13-day-old chick embryos have a maximum width of only about 1.8 mm, insertion of PPy/N electrodes as those used for MAP recording (i.e., with a diameter of around 318 µm) in such a small ventricle is not possible without damaging the heart. Therefore, the stimulation PPy/N electrodes were made from 2.5 mm long, 300 µm wide, and around 50 µm thick PPy/N strips (average thickness of the valleys/hills of the PPy/N strips, see Figure 1e,f). One end of those strips was a sharp cut for being inserted in the ventricle, and the other end was connected to Teflon-coated (76/140 µm) silver leads using silver epoxy to make the bond and nitrocellulose to isolate it. After inserting the PPy/N strips parallel to each other and about 1 mm apart in the ventricle, the leads were connected to a Multistab 235 voltage source (Figure 2). Stimulation was performed using 20 ms width, 100 µV pulses which were delivered manually using a switch and based on visual control after the end of spontaneous MAPs. The resulting electrically evoked MAPs were recorded from the ventricle of nine chick embryo hearts (n = 9) using classic Teflon-coated (200/280 µm) silver electrodes [26] and the recording setup described above.

**Figure 3:**
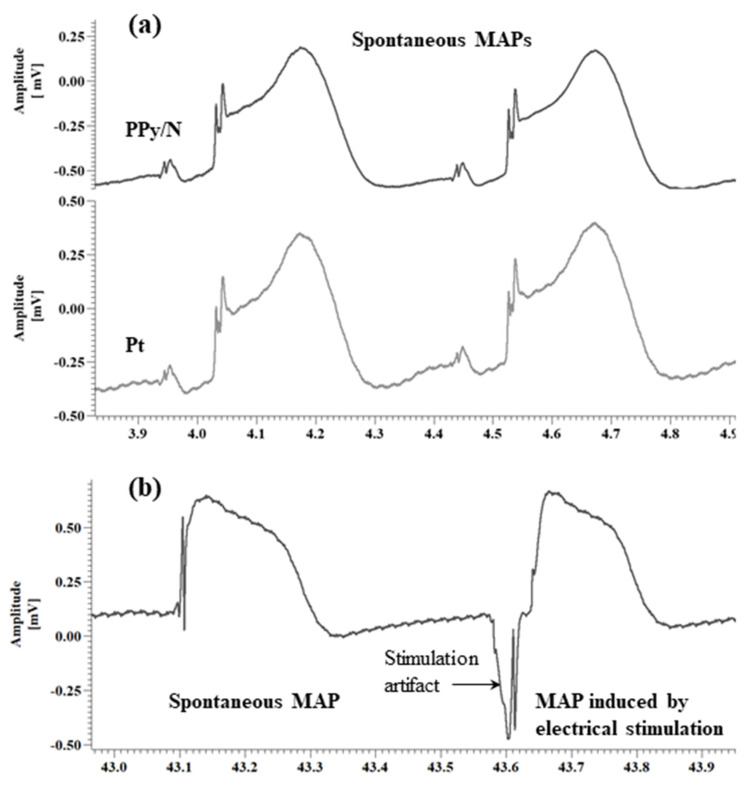
Monophasic action potentials (MAPs) simultaneously recorded from the surface of chick embryo ventricles using PPy/N and Pt electrodes (**a**), and MAPs evoked by electrical stimulation using PPy/N electrodes (**b**). Note: Similar as in Knollmann et al. [26], the morphological difference between the spike-and-dome MAPs from (**a**) vs. the low-plateau MAPs from (**b**) is due to the diameter difference between the electrodes used to record those MAPs, i.e., 300/450 vs. 200/280 µm bare/insulated in (**a**) and (**b**), respectively.

**Figure 4:**
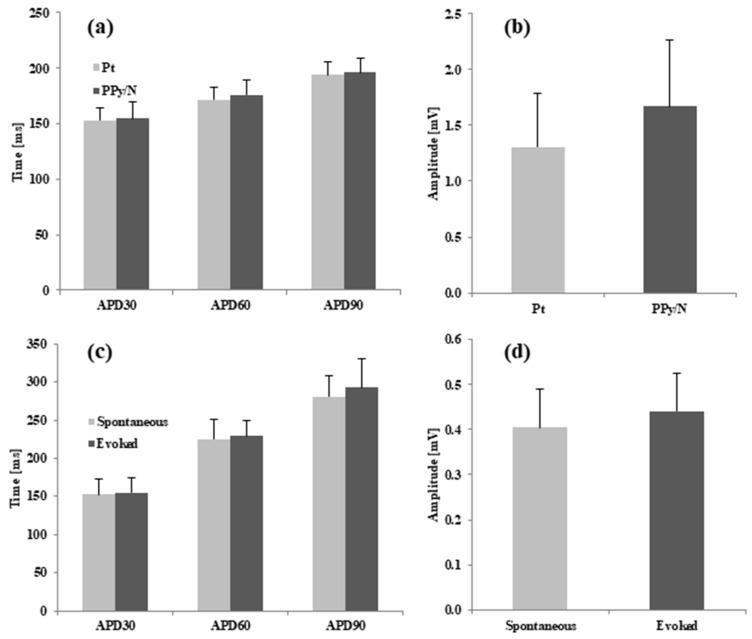
Duration and amplitude parameters of spontaneous MAPs recorded simultaneously with Pt and PPy/N electrodes from the ventricle of 9 chick embryos (**a**,**b**), and spontaneous MAPs vs. MAPs evoked by electrical in the ventricle of 9 chick embryos (**c**,**d**).

*Data analysis.* As indicated by Franz [33], the MAP amplitude was measured between the diastolic baseline and the crest of the MAP plateau phase (Figure 3a). When the crest of the MAP plateau phase could not be determined as in the case of the MAPs evoked by electrical stimulation vs. spontaneous MAPs (Figure 3b), the MAP amplitude was measured according to the method of Knollmann et al. [26] between the diastolic baseline and upstroke peak. As also indicated by Franz [33], the MAP duration (action potential duration, APD) parameters were measured as the time interval from the fastest part of the MAP upstroke and repolarization levels of 30, 60, and 90% of the MAP amplitude, i.e., APD30, APD60, and APD90, respectively. The data were averaged, graphically represented as means + standard errors of the mean (Figure 4), and statistically compared using the paired two-tailed Student’s *t*-test.

### 2.3. Cell Culture Tests

*Cell cultures.* The cell culture tests were performed using human normal fibroblasts (BJ line, CRL-2522), which were purchased from the American Cell Type Culture Collection (ACTCC, https://www.atcc.org–URL accessed on 14 December 2016). The cells were grown in DMEM cell culture medium supplemented with 10% fetal calf serum, 1% L-glutamine, and 1% penicillin-streptomycin in an incubator at 37 °C, 5% CO_2_, and ≥90% humidity. After reaching the exponential phase (i.e., 70% confluency), the cells were transferred to the materials to be tested (i.e., glass coverslips covered with PPy/N and Pt layers) and then left to attach and expand for 24 h. The samples were then fixed using 4% formaldehyde (PFA), washed, and prepared for fluorescence and scanning electron microscopy.

*Fluorescence microscopy analysis.* Cells grown on PPy/N and Pt-covered coverslips were fixed with PFA for 15 min, washed with phosphate-buffered saline (PBS, 3 × 5 min), and stained with Hoechst solution for 2 min in the dark. Finally, cells were washed again with PBS and mounted using a Mowiol-based mountant. Using an Olympus BX51 fluorescence microscope (Microscope Central, Philadelphia, PA, USA), images were taken at 4×, 10×, and 20× magnifications from 3 different points, and the number of cells at those points was quantified using the ImageJ 1.51j8 software [34]. The values were averaged for the two tested materials and statistically compared using the paired two-tailed Student’s *t*-test.

*Scanning electron microscopy analysis.* Samples prepared as described above were washed with PBS (3 × 5 min), then with ddH2O (3 × 5 min), and then were left to dry in air. Samples were then mounted on sample holders with sticky carbon tape and were sputter-coated with a 15 nm layer of Pt/Pd. Images were taken using a Hitachi SU8230 SEM, Hitachi, Tokyo, Japan.

## 3. Results

### 3.1. Morphological and Conductivity Characterization of the PPy/N Coating and Strips

*Morphology of the PPy/N coating and strips.* Figure 1a–f present electron microscopy images of the nylon filaments before and after being coated with PPy/N, thus highlighting the morphological differences and surface changes resulting from the deposition of PPy/N. Figure 1a shows that the uncoated nylon filament exhibits a relatively smooth and shiny surface, whereas Figure 1b reveals the surface irregularities introduced by the abrasive treatment.

The nylon filaments coated with PPy/N exhibited an increased diameter of approximately 320 µm (Figure 1c) compared to the initial 300 µm diameter of the uncoated filaments (Figure 1a). This increase reflects the formation of a homogeneous and continuous PPy layer on the surface of the filament. As shown in Figure 1c,d, the coating encapsulates the nylon filament completely, with no visible discontinuities or detachment zones, which indicates a strong adhesion between the PPy/N layer and the filament. This uniform coverage is essential for ensuring consistent electrical conductivity along the entire length of the PPy/N-coated filament. Further morphological examination at higher magnification (Figure 1e) reveals that the PPy/N layer possesses the typical granular surface structure characteristic of the PPy films, which is associated with enhanced surface area. The thickness of the PPy/N coating was estimated to be approximately 18 µm (Figure 1f), showing that the deposition process allowed a well-controlled and reproducible film growth. The combination of full encapsulation, granular texture, and uniform thickness suggests that the chosen coating parameters are effective in achieving a stable conductive layer, as actually confirmed by the recording and stimulation experiments (see below).

The PPy/N strips exhibited an asymmetric surface morphology, which resulted from the electrochemical deposition process. Thus, as can be seen in Figure 1g, the side of the strip that was in direct contact with the deposition electrode during polymerization presented a relatively smooth surface. This smoothness was due to the constrained growth of the PPy film occurring at the interface with the deposition electrode, where the polymer film conformed closely to the substrate geometry. In contrast, the opposite side of the strip, which was exposed to the electrolyte solution, displayed a pronounced tubercular topography with a “valleys and hills” type structure, which is clearly visible in Figure 1h. As a consequence of this uneven topography, the thickness of the PPy/N strips varied significantly, ranging from approximately 35 µm in the lower regions (valleys) to around 70 µm in the elevated (hills) areas (Figure 1h).

*Conductivity of the PPy/N coating and strips.* The cross-section surface *s* of the PPy/N coating deposited on nylon filaments was 169 × 10^−5^ cm^2,^ and that of the PPy/N strips was 157 × 10^−5^ cm^2^. For the current of 200 µA supplied to the outer probes of the measurement system, a voltage difference *V* of 5 V was measured between the inner probes of that system for both the PPy/N coatings deposited on nylon filaments and PPy/N strips, which resulted in conductivities of 0.27 and 0.29 S/cm for the former and latter, respectively.

### 3.2. Recording of Spontaneous MAPs with Pt vs. PPy/N Electrodes

The spontaneous MAPs simultaneously recorded with Pt and PPy electrodes had identical morphologies (Figure 3a). The average values of APD30, APD60 and APD90 for the MAPs recorded with the Pt vs. PPy/N electrodes were 152.9 ± 34 ms vs. 154.5 ± 45 ms, 171.8 ± 34 ms vs. 176.1 ± 34 ms and 193.7 ± 35 ms vs. 196.3 ± 41 ms, respectively (Figure 4a). None of the differences between the APD30, APD60 and APD90 of the MAPs recorded with the two types of electrodes were statistically significant (*p* > 0.05 for all differences). The average peak-to-peak amplitudes of the MAPs simultaneously recorded with the Pt and PPy/N electrodes were 1.3 ± 0.5 mV and 1.7 ± 0.6 mV, respectively (Figure 4b) and did not differ significantly from a statistical perspective (*p* = 0.3).

### 3.3. Spontaneous MAPs vs. MAPs Evoked by Electrical Stimulation with PPy/N Electrodes

The MAPs evoked by electrical stimulation with PPy/N electrodes and the spontaneous MAPs had almost identical morphologies (Figure 3b). The average values of APD30, APD60 and APD90 for the spontaneous vs. evoked MAPs were 153 ± 61 ms vs. 154.8 ± 57 ms, 225.5 ± 78 ms vs. 229.2 ± 60 ms and 281.1 ± 83 ms vs. 293.4 ± 113 ms, respectively (Figure 4b). None of the differences between the APD30, APD60 and APD90 measured for the spontaneous vs. electrically evoked MAPs were statistically significant (*p* > 0.05 for all differences). The average peak-to-peak amplitudes of the spontaneous MAPs and the MAPs evoked by electrical stimulation with PPy/N electrodes were 0.40 ± 0.1 mV and 0.44 ± 0.1 mV, respectively, and did not differ significantly from a statistical perspective (*p* = 0.3).

### 3.4. Cell Culture Results

**Figure 5:**
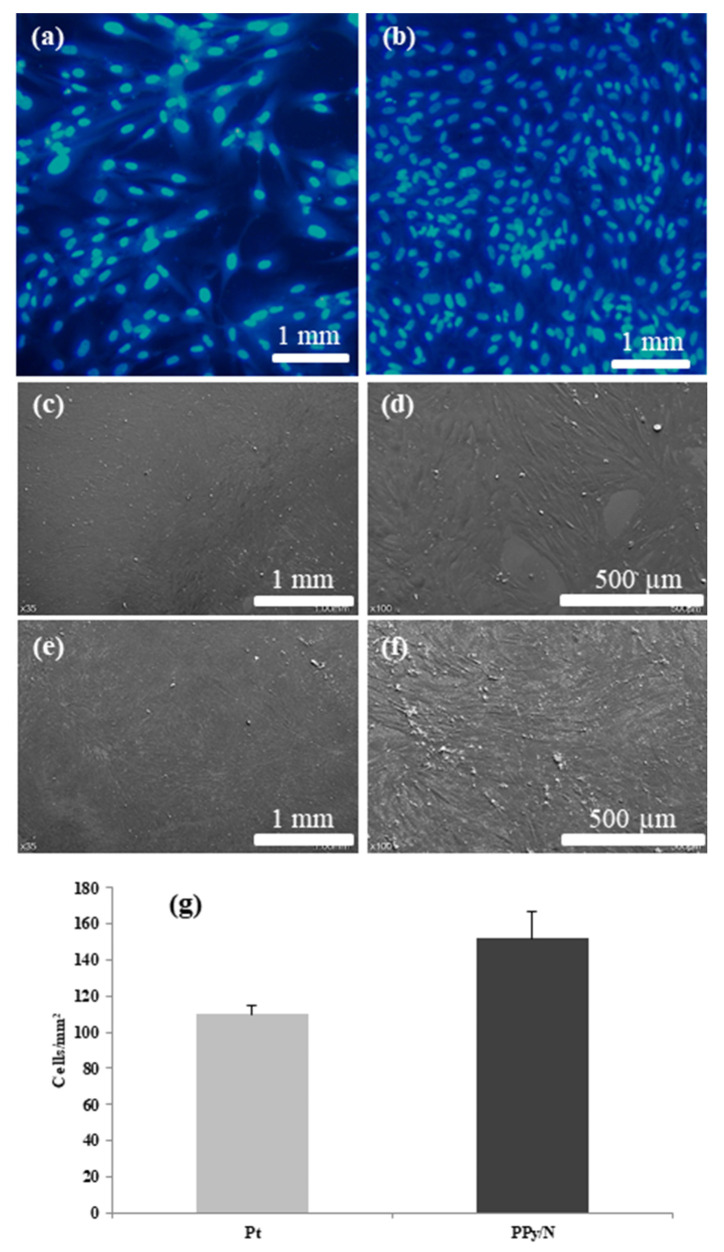
Fibroblasts proliferation on substrates covered with Pt and PPy/N. (**a**,**b**): fluorescence images of fibroblasts grown on Pt and PPy/N substrates, respectively; (**c**–**f**): SEM micrographs of fibroblasts grown on Pt and PPy?n substrates, respectively; (**g**): density of the fibroblasts grown on Pt and PPy/N substrates.

After 24 h of proliferation and growth, the fibroblasts seeded on substrates covered with Pt and PPy/N layers had an average density of 109.6 ± 8.2 and 151.8 ± 25.5 cells/mm^2^, respectively (Figure 5a,b,g). However, that density difference was not statistically significant (*p* = 0.84).

The fibroblast population grown on Pt substrates (Figure 5a,c,d) covered the surface of those substrates almost completely and comprised mostly cells with normally sized nuclei, with about 5–10% of that population consisting of cells with large polyploid nuclei. The cells were flat and expressed their typical furrow morphology with two lamellipodia and numerous fillopodia. In their nuclei, there typically were 2–3 round nucleoli, and in the cytoplasm, a large number of vesicles and bundles of cytoskeleton. While retaining their furrow morphology, the fibroblasts grown on PPy/N substrates appeared to be smaller and thinner than those grown on Pt substrates, with small gaps in between adjacent cells and fewer polyploid cells in the population (Figure 5b,e,f).

## 4. Discussion

The present study shows that PPy/N coatings and free-standing strips can be used to record cardiac action potentials in the form of MAPs almost identical to the MAPs recorded with classical Pt electrodes and to elicit electrically evoked MAPs almost identical to the spontaneous MAPs. This study also suggests that PPy/N has a biocompatibility comparable to that of Pt.

### 4.1. Morphology and Conductivity of the Tested PPy/N Coatings and Strips

As illustrated in Figure 1, the PPy/N coatings deposited on nylon filaments had the typical granular structure of chemically deposited PPy films [35], and the PPy/N strips had the typical tubercular morphology of electrochemically deposited PPy films [36]. Very important from the perspective of fabricating electrodes from PPy, this rough surface of the PPy/N coatings and films creates a surface area much larger than that of a same-size smooth metallic surface. As the recording and stimulation capabilities of implantable electrodes increase proportionally with the surface area of those electrodes [21,22], using PPy/N-coated materials for fabricating electrodes is an advantage from this perspective. The conductivities of 0.27 and 0.29 S/cm of the PPy/N coatings and strips investigated in this study are close to the conductivity range reported for PPy [18] and, as discussed below, resulted in excellent recording and stimulation capabilities of the tested PPy/N coatings and strips.

### 4.2. Recording of MAP Signals with PPy/N Electrodes

As illustrated in Figure 3a, the MAPs recorded with PPy/N coatings deposited on nylon filaments have an identical morphology with the MAPs recorded with classic Pt electrodes placed adjacent to the PPy/N electrodes. This morphological identity is confirmed by the close similarity of the two MAPs concerning their duration parameters (i.e., APD30, APD60, and APD90) and peak-to-peak amplitude (Figure 4a,b). We therefore conclude that thin (around 18 µm, Figure 1f) coatings of PPy/N are as good as Pt regarding their capability to record biological signals. Consequently, if further experiments with chronic implants confirm the long-term stability of that capability, such PPy/N coatings could be used for making non-metallic electrodes softer than those presently used in bioelectronic medicine.

While this study is the first that reports action potentials recorded with PPy coatings deposited on a non-conductive support, our results are consistent with those of previous studies that reported the recording of biological signals with pure PPy electrodes [37] and PPy coatings deposited on metallic electrodes [20,21,22]. Thus, compound cortical action potentials were recorded from the rat cortex with pure PPy electrodes [37], and electrocardiography [20], electromyography [22], and brain signals [21] from mice, rats, and guinea pigs, respectively, with gold electrodes coated with PPy. In addition, similar to our aim to increase the stability of PPy by using the large molecule N as a dopant, Lee et al. used heparine (Hep) [20] and Cui et al. polystyrene sulfonate (PSS) [21] in order to dope PPy coatings deposited on metal electrodes as both molecules are also large and known for being stable [20,21]. Whereas in the study by Lee et al. the Hep molecules were also used to immobilize anti-inflammatory agents and so achieve high-sensitivity and stable electrocardiogram signals with electrodes treated in that manner [20], the second study by Cui et al. reported high-quality compound neural signals recorded with electrodes coated with PPy/PSS from guinea pig cerebellum [21]. In the same direction, in order to stabilize the recording capabilities of PPy layers deposited on metal electrodes, Kim et al. copolymerized PPy and dopamine for this purpose and reported the recording of high-quality electromyography signals using such electrodes [22].

While all the above-mentioned signals seemed robust and stable, the pure PPy electrodes used by Bae et al. required glass capillaries as external supports for being placed in the brain [37] as PPy is fragile [18] and depositing PPy coatings on metal electrodes [20,21,22] do not reduce the rigidity of those electrodes as desired from a mechanical perspective [11,12]. As our results suggest, such problems could be solved using thin PPy/N coatings deposited on soft and flexible, but strong, non-metallic substrates, such as silk [38] or polyvinyl alcohol (PVA) [39] filaments.

### 4.3. MAP Signals Evoked by Electrical Stimulation with PPy Electrodes

As illustrated in Figure 3b, the MAPs that we induced by electrical stimulation using electrodes made from PPy/N films are almost identical to the spontaneous MAPs. The similar duration parameters (i.e., APD30, APD60, and APD90) and peak-to-peak amplitude of the evoked vs. spontaneous MAPs (Figure 4c,d) confirm the morphological identity between the two. We therefore conclude that about 50 µm thick (i.e., the average thickness of valleys/hills, Figure 1h) films of PPy/N could be used to stimulate living tissues. Although this capability could not be demonstrated in this study for PPy/N coatings similar to those used in the recording experiments due to dimension constraints (see Section 2.2.), it is logical to assume that such a stimulation capability would be the same or similar for PPy/N coatings deposited on non-conductive materials, too. If the stability of such a stimulation capability is confirmed in chronic experiments, PPy/N coatings deposited on flexible substrates (e.g., silk or PVA filaments as above) could be used for making non-metallic electrodes softer than those presently used for stimulation in bioelectronic medicine.

While our electrical stimulation experiments were performed on a living excitable organ (i.e., the beating heart), we could not find similar studies with PPy coatings or pure PPy electrodes used to induce action potentials of any kind in animal models or humans. In this regard, the only studies that we could find were studies that showed that various types of cells grow better if seeded on and stimulated with metallic electrodes coated with PPy than if seeded on and stimulated with bare metallic electrodes [20,21,22,23,24].

### 4.4. Biocompatibility of PPy/N Films

As already mentioned, previous studies showed that PPy coatings and films have both good conductivity and biocompatibility [16,17]. While our recording and stimulation results are consistent with the first property (see above), those concerning the PPy/N biocompatibility issue are in line with the latter. In this regard, our experiments showed that the fibroblasts seeded on substrates covered with Pt and PPy/N adhered and developed similarly on both (Figure 5). Meanwhile, although the number of cells counted on the Pt vs. PPy/N substrates did not differ from a statistical perspective, the fibroblasts seeded on PPy/N had a tendency to multiply better than those seeded on Pt (Figure 5). In our opinion, this tendency may suggest a better biocompatibility for PPy/N than for Pt, which is consistent with the observations of others. In this regard, Cui X. et al. reported that rat glial cells grow better on gold electrodes coated with PPy than on bare gold electrodes [40] and Cui S. et al. that human keratinocytes grow better on PPy membranes than on tissue culture plates [19]. Thus, while being in line with the results of previous studies, our results seem to indicate that the biocompatibility of PPy/N is at least as good as that of Pt, i.e., one of the metals that typically are used for the fabrication of implantable electrodes [3].

### 4.5. Study Limitations

The present study indicates that PPy/N coatings and films are capable of conducting biological signals and electrical pulses that can stimulate living organs. Thus, in principle, such PPy/N coatings and films could be used for the fabrication of electrodes able to match the softness of living tissues better than the metallic electrodes if deposited on materials with mechanical properties similar to those of such tissues. However, this latter aspect was not investigated in this study as the tested PPy/N coatings were deposited on nylon filaments only, and the PPy/N films as free-standing strips as imposed by the used animal model. Similarly, while the naphthalin-2-sulfonic acid dopant used in this study has a large molecule and thus should, in theory, conserve the PPY/N conductivity in the aqueous environment from the body, this capability was not investigated in the present study. Meanwhile, while the performed cell culture tests provide clues concerning the biocompatibility of PPy/N, no indication regarding the immune reactions that PPy/N could trigger if implanted in the body can be drawn from this study. Consequently, while this study is a successful first step towards using PPy coatings for implantable electrode fabrication, further experiments for validating such electrodes concerning their electro-mechanical properties, biocompatibility, and long-term stability must be performed in more animal studies before applying this new electrode concept.

## 5. Conclusions

The present work demonstrates that tens of microns of thin PPy/N coatings can be used to record biological signals and stimulate living organs while also facilitating the adhesion and growth of fibroblasts on their surface. This indicates that soft, flexible, and strong substrates such as silk or PVA filaments covered in thin PPy/N coatings could be used for making non-metallic electrodes combining the conduction and biocompatibility capabilities of PPy/N, as demonstrated here with the mechanical properties of the mentioned substrates. While those aspects point to the possibility of using such PPy/N coatings for the fabrication of electrodes that could match the mechanical properties of biological tissues better than the metallic electrodes, this perspective must be validated in further studies on acute and chronic animal models.

## Data Availability

The raw data cannot be made publicly available upon publication because they exist in a format that require a Spike 2 software or higher in order to be visualized, processed and analyzed. However, the data that support the findings of this study are available upon request from the authors.
